# Supplementary material for: Persistence of training-induced visual improvements after occipital stroke
Source: medRxiv. 2024 Oct 24:2024.10.24.24316036. Preprint. [Version 1] doi: 10.1101/2024.10.24.24316036 (PMC11537328; doi:10.1101/2024.10.24.24316036)
Supplement: 1 [file NIHPP2024.10.24.24316036V1-supplement-1.pdf]

medRxiv preprint doi: https://doi.org/10.1101/2024.10.24.24316036; this version posted October 24, 2024. The copyright holder for this preprint (which was not certified by peer review) is the author/funder, who has granted medRxiv a license to display the preprint in perpetuity.
It is made available under a CC-BY 4.0 International license .

Supplementary materials

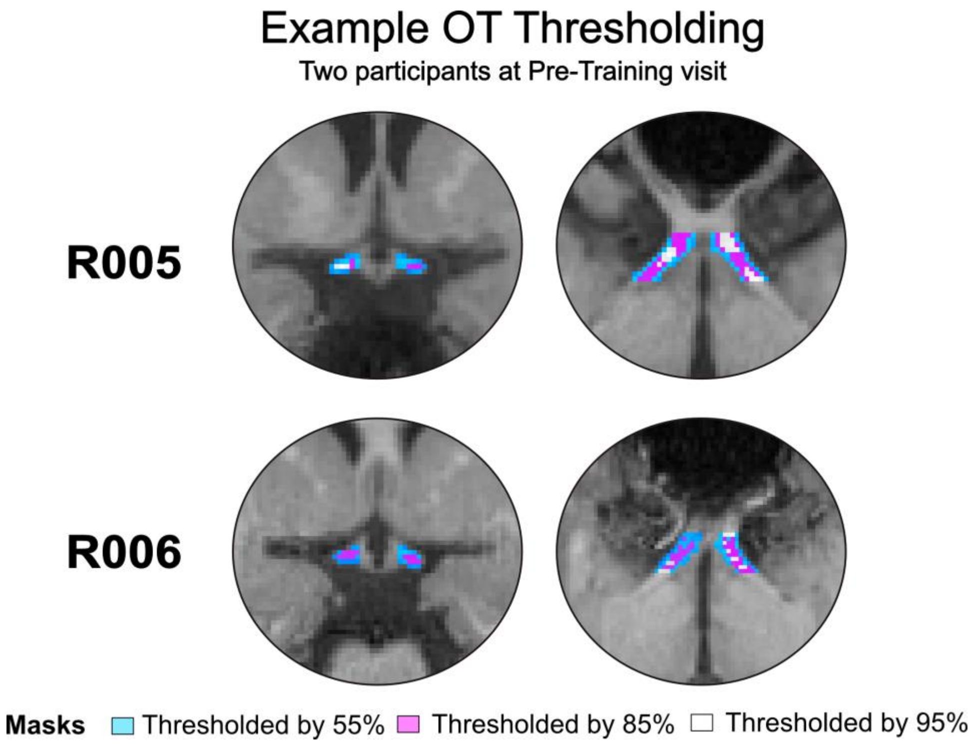

Figure S1. Examples of optic tract structure at higher thresholding. Examples of OT thresholding at 55% (light blue), 85% (pink) and 95% (white). For lower thresholds (55% and 85%) the structure of the optic tract is still maintained. However higher thresholds of 95% results in a patchy and disconnected tract which is likely driven by noise rather than genuine degeneration. For this reason, an 85% threshold was selected.
